# Human DNA decays faster with time than viral dsDNA: an analysis on HPV16 using pathology archive samples spanning 85 years

**DOI:** 10.1186/s12985-021-01529-9

**Published:** 2021-03-29

**Authors:** Sara Nicolás-Párraga, Montserrat Torres, Laia Alemany, Ana Félix, Eugenia Cruz, Silvia de Sanjosé, Francesc Xavier Bosch, Ignacio G. Bravo, the RIS HPV TT study groups

**Affiliations:** 1 Infections and Cancer Laboratory, Cancer Epidemiology Research Program, Catalan Institute of Oncology (ICO), Granvia de L’Hospitalet 199-203, 08908 L’Hospitalet de Llobregat, Spain; 2 Infections and Cancer Unit, Cancer Epidemiology Research Program, Catalan Institute of Oncology (ICO), Barcelona, Spain; 3 Bellvitge Institute of Biomedical Research (IDIBELL), Barcelona, Spain; 4 Pathology Unit, Portuguese Institute of Oncology Francisco Gentil (IPO Lisbon), Lisbon, Portugal; 5 Pathology Unit, Portuguese Institute of Oncology Francisco Gentil (IPO Coimbra), Coimbra, Portugal; 6 Sexual and Reproductive Health, PATH, Seattle, USA; 7 CIBER in Epidemiology and Public Health (CIBERESP), Madrid, Spain; 8 Biomedical Research Networking Centre On Cancer (CIBERONC), Madrid, Spain; 9 Universitat Oberta de Catalunya, Barcelona, Spain; 10 Laboratory MIVEGEC (CNRS IRD Univ Montpellier), French National Center for Scientific Research (CNRS), Montpellier, France; 11 Center for Research On the Ecology and Evolution of Diseases (CREES), Montpellier, France

**Keywords:** Human Papillomavirus 16, Invasive cervical carcinoma, Formalin-Fixed Paraffin Embedded, Viruses, DNA, Degradation, Integrity, Sample storage, Stability

## Abstract

**Background:**

Quality of the nucleic acids extracted from Formalin Fixed Paraffin Embedded (FFPE) samples largely depends on pre-analytic, fixation and storage conditions. We assessed the differential sensitivity of viral and human double stranded DNA (dsDNA) to degradation with storage time.

**Methods:**

We randomly selected forty-four HPV16-positive invasive cervical cancer (ICC) FFPE samples collected between 1930 and 1935 and between 2000 and 2004. We evaluated through qPCR the amplification within the same sample of two targets of the HPV16 *L1* gene (69 bp, 134 bp) compared with two targets of the *human tubulin-β* gene (65 bp, 149 bp).

**Results:**

Both viral and human, short and long targets were amplified from all samples stored for 15 years. In samples archived for 85 years, we observed a significant decrease in the ability to amplify longer targets and this difference was larger in human than in viral DNA: longer fragments were nine times (CI 95% 2.6–35.2) less likely to be recovered from human DNA compared with 1.6 times (CI 95% 1.1–2.2) for viral DNA.

**Conclusions:**

We conclude that human and viral DNA show a differential decay kinetics in FFPE samples. The faster degradation of human DNA should be considered when assessing viral DNA prevalence in long stored samples, as HPV DNA detection remains a key biomarker of viral-associated transformation.

**Supplementary Information:**

The online version contains supplementary material available at 10.1186/s12985-021-01529-9.

## Background

In pathology routine most biopsies and surgical samples are formalin fixed paraffin embedded (FFPE) to preserve tissue structure and cellular morphology. Biobanks and FFPE sample collections constitute valuable sample sources because they allow to create and maintain well-archived, extensive, easy to handle and relatively inexpensive sample repositories to perform large histological and molecular retrospective studies [1–3].

For molecular epidemiology studies, the information targeted is usually nucleic acid sequencing, but tissue preservation in paraffin blocks is variable and depends on a number of factors in the different steps of the process that affect quantity and quality of the extracted nucleic acids [4, 5]. One of the most important and studied factors is the type of the fixative chosen for the fixation step, with formalin being the commonest one. Formalin fixation occurs through the formation of methylene bridges between the aldehyde group in the fixative and the amino groups in nucleic acids and proteins [6]. Over-fixation or the use of a fixative molecule with multiple active groups, *e.g.* glutaraldehyde, hardens the tissue by cross-linking multiple molecules and can induce physical fragmentation of the nucleic acids during extraction [7, 8]. If unbuffered, the aldehyde group in formalin can undergo chemical disproportionation, so that two molecules of formaldehyde yield one molecule of methanol and one molecule of formic acid. The exposure of DNA to methanol and formic acid can lead to DNA depurination and strand breaks [6, 9]. Overall, DNA damage induced by fixation conditions can hinder downstream applications based on amplification techniques [10, 11].

Other important factor for the effects of the fixation process on nucleic acid quality is the duration of storage period and in this regard the literature is contradictory. While certain studies describe that the use of FFPE specimens stored for several years (i.e. samples stored between 10 to 20 years)  has only minor effects on subsequent DNA analysis [12, 13], other authors show that the length of PCR-amplified fragments and whole genome amplified-fragments decreases with storage time even when coupled with optimized DNA extraction procedures [14].

Persistent infection by oncogenic Human Papillomaviruses (HPVs) is the major risk factor for the development of cervical cancer and is associated with most anal and vaginal cancers, as well as with a significant fraction of vulvar, penile and oropharyngeal cancers [15]. Most assays for screening and diagnosis of HPVs-related diseases rely on the detection of viral nucleic acids and include amplification steps, often using consensus or multiplexed PCR [16]. Although fixation and storage affect the efficiency of consensus PCR assays for HPVs detection, FFPE specimens remain a crucial source for molecular epidemiology purposes when fresh clinical material is unavailable -which is often the case- making it possible to perform large retrospective studies correlating molecular features with therapeutic response and clinical outcome [17–21].

This work was designed to evaluate the differential impact of time storage on the quality, quantity and degradation of viral and human DNA employing a quantitative PCR on FFPE invasive cervical cancer samples HPV16 single infected that had been archived for 15 and 85 years.

## Methods

### Sample selection

Samples used in this study belonged to a repository including FFPE of primary invasive squamous cell cervical cancer tissues from hospital pathology archives. Among all centers that had supplied samples for the historic retrospective study, we focused on the one single institution that maximized time span [21]. By using a single source, we intended to homogenize sample treatment and procedures. The procedures employed for FFPE sectioning, DNA purification and HPV DNA detection and genotyping have been previously described [21–23]. Samples had been formalin fixed under standard conditions, using a 10% v/v of a formaldehyde (gas) saturated solution in water, corresponding to a final concentration of 3.7–4.0% w/v formaldehyde. As it became a common practice in anatomopathology departments over the world, from late 1990s this fixative solution was prepared using phosphate buffer saline, to prevent formaldehyde disproportionation that can occur during long time storage if unbuffered. Briefly, four 5-µm paraffin sections were systematically obtained from each block. The first and last sections were used for histopathological assessment, and the second and third sections were used for backup and analysis of HPVs DNA, respectively. DNA was released by incubation of the tissue sections for 16 h at 56 °C with 250 µL buffer (10 mg/mL proteinase K, 50 mM Tris–HCl, 1.0 mM EDTA, 0.5% Tween 20, pH 8.0), followed by 10 min at 95 °C to inactivate the protease. HPV DNA detection were performed through PCR-DEIA using a consensus primers (SPF_10_) that target the *L1* gene and amplify a 65 base pairs (bp) fragment. HPV DNA genotyping of positive samples was performed employing LiPA25 system (version 1; DDL Diagnostic Laboratory, Rijswijk, The Netherlands) [24, 25]. Samples were stored at − 80 °C.

The selection of the samples for this study was restricted to invasive cervical cancer (ICC) cases having tested positive exclusively for the presence of HPV16 DNA, this way reducing the possible competition between different HPVs for the primers during amplification step. We chose fifty HPV16-positive ICC samples, 25 samples collected between 1930 and 1935, i.e. archived for 80–85 years and referred to as 85 years storage, and 25 samples between 2000 and 2004, i.e. archived for 11–15 years and referred to as 15 years storage. We double-checked as far as possible the actual nature of the fixative used in these samples treated in the 30s. Exhaustive searches confirmed that 19 of these samples had actually been fixed using formalin solution. However, three of the samples had been treated with the so-called Zenker solution as fixative, a mixture using heavy cations in an acid environment [22] while for three of the samples we could not elucidate with certainty the fixative. For this reason, and given that we could not find any additional sample fulfilling the criterion of single-infection by HPV16 from this period, we decided to perform the laboratory analyses on all 50 samples, but to run the statistical analyses only on using the 19 samples from the 30s for which we can assure they have been fixed in formalin. Nevertheless, we present the results for the full comparison. All results are similar both in trend as well as in significance level of the corresponding comparisons.

### Quantitative PCR assay

Primers were designed through Primer3 plus (http://www.bioinformatics.nl/cgi-bin/primer3plus/primer3plus.cgi/) to amplify fragments no longer than 200 bp with similar amplicon sizes (differences of ± 15 bp), melting temperatures (around 64 °C), and GC content (50%). For the detection of HPV16 DNA, we targeted the *L1* gene as the most conserved open reading frame at the nucleotide level within Papillomaviruses [26]. For the detection of human DNA we chose *tubulin*-β gene, which had been used for quality control purposes of the original FFPE repository [19]. For the *tubulin*-β gene we verified the conservation of the primer targets in the available Human 1000 Genomes (TUBB, hg38 chr6:30,720,352–30,725,422), and the absence of off-target amplification by means of primer-BLAST (https://www.ncbi.nlm.nih.gov/tools/primer-blast/). PCR systems were designed to respectively amplify two fragments of 69 bp and 134 bp within *L1*, and 65 bp and 149 bp in *tubulin*-β, employing for each gene a common forward primer and two different reverse primers. Primer sequences and amplicon sizes are shown in Table 1.

**Table 1:** Primers sequences for human and viral PCR amplification

Primers	Sequence 5′– 3’	Amplicon Size
TUBB-F	TCCTCCACTGGTACACAGGC	—
TUBB-R1	CATGTTGCTCTCAGCCTCGG	65 bp
TUBB-R2	CTCCTCTTCGGCCTCCTCAC	149 bp
HPV16_L1-F	AATAGGGCTGGTRCTGTTGG	—
HPV16_L1-R1	TGCAGTAGACCCRGAGCCTT	69 bp
HPV16_L1-R2	ATTTGGGCATCAGAGGTAACCAT	134 bp

To maximize and improve the DNA yield from the stored samples, a recovery protocol was applied before amplification that included a pre-heat step of 60 °C during 48 h to facilitate the release of DNA adsorbed to the plastic walls of the tubes. The concentration of DNA was measured with Qubit dsDNA HS Assay kit (Invitrogen, Life Technologies, CA, USA) on a Qubit 3.0 Fluorometer (Invitrogen, Life Technologies, CA, USA) according to manufacturer’s instructions.

SYBR-green based quantitative PCR (qPCR) was performed in 20 µL reaction mix containing FastStart Essential DNA Green Master (Roche Molecular Systems, Branchburg, NJ, USA), 0.3 µM of each forward and reverse primer, and 2 µL of DNA. qPCR amplification was performed using the LightCycler® 96 Real-Time PCR System (Roche Molecular Systems) programmed for 10 min at 95 °C, followed by 40 cycles of 10 s at 95 °C, 10 s at 64 °C and 10 s at 72 °C. After data acquisition, the cycle threshold value was calculated by determining the point at which the fluorescence reached the threshold limit of 0.2. Following amplification, melting curve analysis was performed to assess the nature of the PCR product using a melting program with an increase of 2.2 °C/s from 65 °C to 97 °C. The standard curve used for quantification of *tubulin*-β was performed with 7-fold serial 1:5 dilutions of human genomic DNA (Roche Molecular Systems) starting with 0.2 mg/mL. For *tubulin*-β quantification we considered that one cell contains approximately 6 pg of DNA and that a diploid cell contains two copies of *tubulin*-β. For *L1* quantification, the standard curve was performed using 7-fold serial 1:5 dilutions of an international standard for HPV16 (NIBSC, London, UK). According to manufacturer’s instructions HPV16 plasmid stock was 1.0 × 10^7^ genome equivalents/mL. All samples and controls were tested in technical triplicates, and were considered positive for the analysis when the quantification cycle (Cq) value for either *tubulin*-β or *L1* was below 35 cycles. Quantification of human and viral DNA was expressed as copies *per* µL.

### Statistical analyses

Analyses were conducted with R statistical package (version 3.2.5). We calculated Exact McNemar's test to evaluate the discordant results. We also used Fisher’s exact tests for the comparison of presence/absence of viral and human PCR amplification. For quantitative variables, comparisons were performed using the Wilcoxon rank-sum in the case of unpaired values and the Wilcoxon signed-rank test in the case of paired values. All tests were two-tailed and the cut off value for significance was set at *p* < 0.05.

## Results

### Overall concentration of DNA retrieved from FFPE samples.

The DNA concentration in samples stored for 15 years was around 25% higher than in those stored for 85 years. Respective median values were 5.2 ng/µL (IQR: 3.2–11.0) and 1.2 ng/µL (IQR: 0.6–1.6) (Wilcoxon rank sum test, *p* = 2.6*10^–6^).

### Differential quality of viral and human DNA retrieved from FFPE samples with storage time.

We analyzed first the differential amplification ability of long and short fragments in human and viral DNA between samples with the same storage time. In all (100%) samples stored for 15 years, we could successfully amplify the two fragments sizes for both human and viral DNA. In the set of samples archived for 85 years, for both human and viral DNA, when the long amplicon was generated the corresponding short amplicon was also retrieved. In samples stored for 85 years, overall congruence between the amplification results obtained for human and for HPV16 DNA (Table 2) was low (21% concordance). This weak concordance reflected that human DNA amplification of the long fragment was less likely than that of the short fragment (respectively 2/19 *vs* 17/19; Exact McNemar test, *p* = 6.1*10^–5^), while for viral DNA this difference was not observed (12/19 *vs* 16/19; Exact McNemar test, p = 0.125).

**Table 2:** Score based on amplified fragments in the set of samples archived for 85 years

	No. of samples amplified on human *tubulin*-β gene			
	Negative	Positive (65 bp)	Positive (149 bp)^†^	Total
No. of samples amplified on HPV16 *L1* gene				
Negative	0	3	0	3
Positive (69 bp)	2	2	0	4
Positive (134 bp)^‡^	0	10	2	12
Total	2	15	2	19

bp: base pairs

^†^All samples named “149 bp” were also positive for both 65 bp and 149 bp amplicons

^‡^ All samples named “134 bp” were also positive for both 69 bp and 134 bp amplicons

We analyzed the differential amplification ability of the same DNA target in different samples with regards to the storage time. We did not observe differences between samples stored for 85 and for 15 years in the potential for generating the short amplification product, neither for human DNA (17/19 *vs* 25/25, Fisher’s Exact test; *p* = 0.18) nor for HPV16 DNA (16/19 *vs* 25/25, Fisher’s Exact test; *p* = 0.07). In contrast, viral and human DNA responded very differently to FFPE to long-term storage regarding the amplification of longer products. Human DNA amplification potential dropped significantly: 25/25 after 15 years and 2/19 after 85 years storage (Fisher’s Exact test; *p* = 2.5*10^–10^) (Table 2). The relative amplification of the long amplicon was thus nine times higher for samples with 15 years of storage than for samples with 85 years of storage (Risk ratio: 9.5; CI 95% 2.6–35.2). Regarding viral DNA instead, the decrease in amplification of the long product with time was far less important: 25/25 after 15 years *vs* 12/19 after 85 years storage (Fisher’s Exact test; *p* = 1.3*10^–3^). In this case, the effect of storage time for the relative amplification was lesser (Risk ratio: 1.6; CI 95% 1.1–2.2). Our results show therefore that FFPE long-term storage decreased qualitatively the ability to recover longer DNA fragments by means of amplification, and that the impact of DNA degradation was more important for human than for HPV16 DNA.

### Differential quantification of viral and human DNA retrieved from FFPE samples with storage time.

**Fig. 1:**
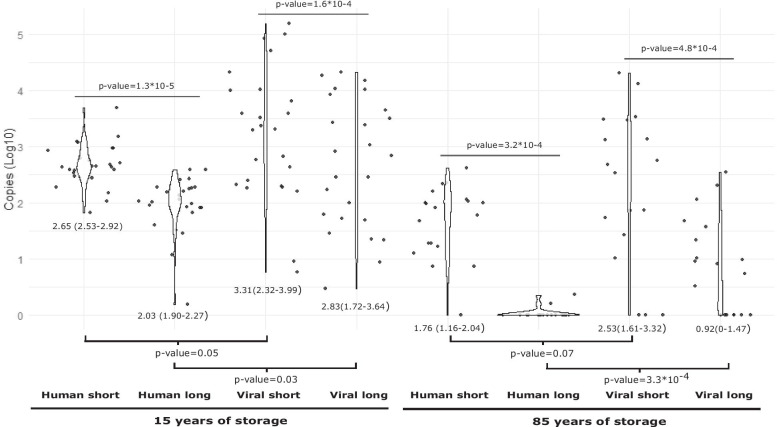
Violin plots comparing the distribution of the quantification values by gene, amplicon size and storage time including only the subset of samples fixed in formalin. For each period of time, median values of copies/µL (depicted as Log_10_) *per* amplicon either between host and virus or for the two different amplicons within host and within viruses are compared by means of a Wilcoxon Mann–Whitney test. For each comparison, p-values for the null-hypothesis of non-different median values between the corresponding distributions are indicated. Fragments are represented as follows: “Human short” to represent 65 bp *tubulin*-β gene amplicon; “Human long” to represent 149 bp *tubulin*-β gene amplicon; “Viral short” to represent 69 bp *L1* gene amplicon and “Viral long” to represent 134 bp *L1* gene amplicon. Each period of time is represented below fragments

We have further quantitatively validated the results on the differential potential for amplification by means of qPCR. In all samples, quantification values for the short amplification target and for the long target were different for both viral and human DNA (for 15 years storage, Wilcoxon signed rank test, *p* = 1.3*10^–4^ and *p* = 1.3*10^–5^, respectively for viral and human targets; for 85 years storage, Wilcoxon signed rank test, *p* = 4.8*10^–4^ and *p* = 3.2*10^–4^, respectively for viral and human targets). In both cases, the short fragment was estimated to be around four times more present than the long fragment. Similarly, for samples archived for 85 years the values obtained using the short amplicon were different, approximately forty times more present, compared with the ones obtained when was amplified the long amplicon, both for human and HPV16. The results revealed a differential DNA quantification for human and HPV affected by the length of the amplicon, both in samples archived for 15 and for 85 years (Figs. 1 and Additional file 1: Fig. S1).

## Discussion

Archived FFPE specimens are an invaluable source for molecular biological analyses, molecular epidemiology studies and/or identification of biomarkers. Tissue preservation in paraffin blocks is variable and dependent of multiple factors that influence nucleic acid integrity, potentially affecting the results for long-term retrospective analyses. Considering HPVs and the associated cancers, some studies have assessed the usefulness of FFPE samples and the different viral detection efficiency regarding distinct techniques [3, 24], while other have examined whether the storage period has significant effect on DNA, RNA or protein retrieval, however, focusing solely in human macromolecules [2]. Additional research studied DNA amplification using different amplicon sizes [13], albeit without considering the different origin of DNA, i.e. human or viral DNA. In the present work we have tried to combine all these approaches to provide a complete picture on the quality and quantity of viral and human DNA retrieved from samples stored for 85 and 15 years, and using two sets of different sized amplicons (*ca*. 70 bp and *ca*. 150 bp) implemented in different qPCR systems on invasive cervical FFPE samples exclusively containing HPV16 as viral agent. Our results show that longer fragments of HPV dsDNA can be amplified from 80-years old FFPE samples compared with human dsDNA. Differential amplification could reflect increased chemical stability of the episomal, often supercoiled viral DNA and/or be related to the large number of copies *per* infected cell of viral DNA either in form of integrated concatemers or of episomes, as discussed below.

Our qualitative results for human and viral DNA detection describe higher efficiency and quality of amplification with primers amplifying shorter fragments, in line with previous reports [25, 27]. Álvarez-Aldana and coworkers amplified human and viral DNA from a set of FFPE cervical tissues stored for six years. They worked with 209 and 110 bp fragments in human β-*globin* gene and with 142 and 96 bp amplicons in HPV16 *L1* and *E6* genes respectively, and they observed higher quality of detection in human DNA compared with HPV DNA [25]. The results from this study contrast with ours, but the origin for the lack of concordance lies probably at the primers used for viral amplification and at the ill-defined set of samples. Indeed, for the small HPV amplicon these authors used the GP5+/6+ set of generic primers [28], suited for a broad detection of HPVs but not specific enough for assessing sensitivity, and definitely not for a type-specific qPCR, which has been our choice for primer design. For the long HPV amplicon the authors used the TS16 primers, targeting HPV16 [25], but the actual genotype that could have been detected in the lesions had never been identified in forehand, so that a negative result is not necessarily informative. In our case, we have exclusively worked with samples containing HPV16, and our primers were designed with the same degree of specificity for the human and for the viral targets. In our hands, results from the PCR system on the human *tubulin*-β target are more dependent on the fragment size than on the viral *L1* gene. The same amplification pattern for human DNA depending on amplicon size was observed by Nakayama and coworkers, who amplified different targets of the human *gapdh* gene from paraffin samples (spanning from ≈300 bp to ≈1350 bp) and reported higher proportion of short amplicons, suggesting less amplification efficiency for larger fragment sizes [29].

Regarding differential amplification with storage time, Ademà and coworkers studied FFPE DNA integrity of certain human genes between different storage periods and observed that DNA integrity decreased in FFPE samples stored with eight years of difference [1]. Our results spanning a longer period of time point in the same direction: with longer storage time, human DNA quality, as estimated by qPCR in terms of amplifiable length, is lower and only a minority of the analyzed samples remained equally amplifiable for short and long fragments. Furthermore, we observed that the rates of amplification for short fragments either human or HPV are similar in spite of the time of storage (85 years of storage *vs* 15 years of storage), whereas for large fragments, we observed different amplification time trends, being more appreciable in human DNA than in viral. Specifically, whereas quality of human DNA was nine times more prone to amplify for samples storage 15 years than 85 years, viral DNA only was less than two times more prone. According to these time-differential PCR-fragments rates of amplification observed, we suggest that PCR systems designed to amplify DNA fragments ≥ 150 bp from FFPE samples stored more than 70 years, may result in false negatives due to the decreased sensitivity of the PCR system.

Herráez-Hernández and coworkers compared different HPV genotyping systems that include an initial PCR step of different fragments lengths [30]. They concluded that there are differences in sensitivity rates as function of amplicon size, suggesting that systems based on the amplification and genotyping of short HPV DNA fragment size, for instance INNO-LiPA® HPV Genotyping (Fujirebio, Belgium) that includes the amplification of 65 bp fragment through consensus primers SPF_10_, present higher sensitivity to detect HPV DNA in FFPE samples than other techniques that work with larger DNA fragment, *e.g.* HPV2 CLART (Genomica, Spain) or Linear Array Genotyping test (Roche Molecular Systems), both detecting a 450 bp amplicon size. The same amplification pattern was described by Martró and coworkers comparing two HPV assays (INNO-LiPA® HPV Genotyping and *F*-HPV typing™), which amplify fragments of respectively 158 bp and 484 bp in length in the *E6/E7* region, and reporting higher positivity for the approach using shorter amplicon size for initial steps [31]. In our work, we have additionally compared human and HPV PCR systems, in order to assess dependence of the system performance. We observe that human and viral PCR systems perform differently for the same sample. We confirm that in FFPE samples, especially in those with a prolonged storage, amplification based-genotyping assays should use amplicons of around 60–70 bp in order to be highly sensitive. Furthermore, we propose that internal controls should not only be focused on human DNA, and if so, they should use amplicons of similar 60–70 bp length, as we have observed significant differences of PCR amplification with the *tubulin*-β gene, not detected for HPV DNA fragments.

Relating to the quantifying methodology implemented in our work, Nakayama and colleagues suggested that qPCR reflects more accurately the degree of DNA fragmentation than other techniques (*e.g.* UV or fluorescence spectroscopy) [28]. Thus, in our research we analyzed the trends intra and inter storage time-periods of the quantification values, and we further explored the variance of these values when stratifying not only by time, but also by the DNA nature and the amplicon size. For all comparisons for the same storage time, amplicon size influences the quantification values, in all cases we obtained higher values of quantification for short fragments compared with large. Dietrich and coworkers, developed a semi-nested qPCR system (a unique forward primer and different reverse primers) for the *pitx2* human DNA gene covering 13 amplicon lengths ranging from 200 to 850 bp to be employed in FFPE samples collected between 1992 and 2011. These authors did not observe viral load variation or qPCR inhibition, reporting Cq values of 40 when increasing the fragment length above 200/250 bp [10]. Dal Bello and colleagues quantified the viral load employing a qPCR system with the SPF_10_ consensus primers on 173 FFPE samples collected in three periods (1985–87; 1995–97 and 2005–07), and they observed that HPV titers did not present differences attributable to duration in recently stored samples [32]. We propose that for studies in which the goal is to quantify human (targeting *tubulin*-β) or HPV DNA (targeting *L1*) in FFPE samples recently stored or stored ≥ 15 years through qPCR, the amplicon sizes should to be considered as one of the main variables to avoid possible under-estimation of the viral load or copies/µL. The obtained data could be helpful in deciding the design of qPCR amplification on FFPE samples with storage time below 15 years.

This study is subject to some a number of limitations. First, the paraffin employed for the embedding process has moved along the years to lower melting temperatures and consequently, the DNA purification could be differentially impacted by an inefficient releasing in the initial steps in the samples archived for 85 years versus those archived for 15 years. In addition, the buffered formalin used beyond 2000 has shown higher PCR efficiency rates compared to unbuffered formalin. The more recent cases were collected from Portuguese Institutes of Oncology *Francisco Gentil* of Coimbra and Lisbon. In spite of the standardization of the routine sample processing in the institution, there could exist slight variations between both cities that could affect the preservation of the DNA in the tissues. A second limitation concerns the targeted genes: only one gene *per* genome, viral and human. Our results of an increased lack of detection for human DNA with the time of storage suggest that it could be advisable to test multiple human gene to assess the suitability of a sample for epidemiological studies. Third, we have developed for this study a novel qPCR system. It was not our aim to exhaustively describing the sensitivity of this amplification system to detect HPV infections, and we have thus restrained ourselves to samples that had already been tested positive by using the very sensitive and well characterized SPF_10_-DEIA-LiPA25 algorithm. Indeed two of the samples from the 1930s that had tested positive with the SPF_10_ primers were negative with our novel primer set. Fourth, we have used in our study exclusively invasive cervical cancer samples, with the aim of standardizing the input material. Given that the HPV16 DNA in a cervical cancer lesion may be found integrated, as an episome or as a mixture of both, there may be certain degree of heterogeneity in the nature of the target DNA. Nevertheless, our study aims at providing a reference for epidemiological studies about causality as inferred from the association between disease and pathogen DNA recovery. Finally, our study was performed with a small sample size, obviously limited by the availability of appropriate samples from the 1930s. We would obviously have liked to work with a larger sample set to increase statistical power. However, it was complicated to find additional FFPE samples fulfilling the criteria for inclusion in the study, largely because of the fixative considerations described above.

## Conclusions

We conclude that for FFPE samples with a prolonged storage, time affects the performance of both viral and human PCR systems generally decreasing amplification viability for amplicons above ≥ 150 bp. This DNA degradation with storage time is more evident for human DNA, which seems to present a faster decay kinetics than viral dsDNA. We hypothesize that the episomal nature of viral DNA may underlie this enhanced robustness of HPV16 DNA to chemical degradation. Overall, the differential degradation behavior of human and viral DNA should be considered during the experimental design when assessing prevalence of viral DNA in ancient samples to prevent biases.

## Supplementary Information

**Additional file1 Fig S1**. Violin plots comparing the distribution of the quantification values by gene, amplicon size and storage time including all samples. For each period of time, median values of copies/µL (depicted as Log_10_) *per* amplicon either between host and virus or for the two different amplicons within host and within viruses are compared by means of a Wilcoxon Mann–Whitney test. For each comparison, p-values for the null-hypothesis of non-different median values between the corresponding distributions are indicated. Fragments are represented as follows: “Human short” to represent 65 bp *tubulin*-β gene amplicon; “Human long” to represent 149 bp *tubulin*-β gene amplicon; “Viral short” to represent 69 bp *L1* gene amplicon and “Viral long” to represent 134 bp *L1* gene amplicon. Each period of time is represented below fragments.

## Data Availability

All data generated or analyzed during this study are included in this article and its additional file.

## Abbreviations

HPV: Human papillomavirus
FFPE: Formalin fixed paraffin embedded
ICC: Invasive cervical carcinoma
qPCR: Quantitative PCR
IQR: Inter quartile range

## References

[1] Ademà V, Torres E, Solé F, Serrano S, Bellosillo B (2014). Paraffin treasures: do they last forever?. Biopreserv Biobank.

[2] Kokkat TJ, Patel MS, McGarvey D, LiVolsi VA, Baloch ZW (2013). Archived formalin-fixed paraffin-embedded (FFPE) blocks: a valuable underexploited resource for extraction of DNA, RNA, and protein. Biopreserv Biobank.

[3] Morshed K, Polz-Dacewicz M, Szymański M, Smoleń A (2010). Usefulness and efficiency of formalin-fixed paraffin-embedded specimens from laryngeal squamous cell carcinoma in HPV detection by IHC and PCR/DEIA. Folia Histochem Cytobiol.

[4] Gilbert MTP, Haselkorn T, Bunce M, Sanchez JJ, Lucas SB, Jewell LD (2007). The isolation of nucleic acids from fixed, paraffin-embedded tissues-which methods are useful when?. PLoS One..

[5] Srinivasan M, Sedmak D, Jewell S (2002). Effect of fixatives and tissue processing on the content and integrity of nucleic acids. AmJPathol..

[6] Howat WJ, Wilson BA (2014). Tissue fixation and the effect of molecular fixatives on downstream staining procedures. Methods..

[7] Douglas MP, Rogers SO (1998). DNA damage caused by common cytological fixatives. Mutat Res - Fundam Mol Mech Mutagen..

[8] Masuda N, Ohnishi T, Kawamoto S, Monden M, Okubo K (1999). Analysis of chemical modification of RNA from formalin-fixed samples and optimization of molecular biology applications for such samples. Nucleic Acids Res..

[9] Bonin S (2003). PCR analysis in archival postmortem tissues. Mol Pathol..

[10] Dietrich D, Uhl B, Sailer V, Holmes EE, Jung M, Meller S (2013). Improved PCR performance using template DNA from formalin-fixed and paraffin-embedded tissues by overcoming PCR inhibition. PLoS One..

[11] Taga M, Eguchi H, Shinohara T, Takahashi K, Ito R, Yasui W (2013). Improved PCR amplification for molecular analysis using DNA from long-term preserved formalin-fixed, paraffin-embedded lung cancer tissue specimens. Int J Clin Exp Pathol..

[12] Jaremko M, Justenhoven C, Abraham BK, Schroth W, Fritz P, Brod S (2005). MALDI-TOF MS and TaqMan® assisted SNP genotyping of DNA isolated from formalin-fixed and paraffin-embedded tissues (FFPET). Hum Mutat..

[13] Talaulikar D, Shadbolt B, McNiven M, Dahlstrom JE (2008). DNA amplification from formalin-fixed decalcified paraffin-embedded bone marrow trephine specimens: does the duration of storage matter?. Pathology [Internet]..

[14] Bass BP, Engel KB, Greytak SR, Moore HM (2014). A review of preanalytical factors affecting molecular, protein, and morphological analysis of formalin-fixed, paraffin-embedded (FFPE) tissue: how well do you know your FFPE specimen?. Arch Pathol Lab Med.

[15] de Martel C, Plummer M, Vignat J, Franceschi S (2017). Worldwide burden of cancer attributable to HPV by site, country and HPV type. Int J Cancer..

[16] Ikenberg H (2014). Laboratory diagnosis of human papillomavirus infection. Curr Probl Dermatol [Internet]..

[17] Alemany L, Cubilla A, Halec G, Kasamatsu E, Quirós B, Masferrer E (2016). Role of human papillomavirus in penile carcinomas worldwide. Eur Urol.

[18] Alemany L, Saunier M, Tinoco L, Quirós B, Alvarado-Cabrero I, Alejo M (2014). Large contribution of human papillomavirus in vaginal neoplastic lesions: A worldwide study in 597 samples. Eur J Cancer.

[19] Alemany L, Saunier M, Alvarado-Cabrero I, Quirós B, Salmeron J, Shin H-R (2015). Human papillomavirus DNA prevalence and type distribution in anal carcinomas worldwide. Int J Cancer.

[20] Castellsagué X, Alemany L, Quer M, Halec G, Quirós B, Tous S (2016). HPV involvement in head and neck cancers: comprehensive assessment of biomarkers in 3680 patients. J Natl Cancer Inst..

[21] de Sanjose S, Quint WG, Alemany L, Geraets DT, Klaustermeier JE, Lloveras B (2010). Human papillomavirus genotype attribution in invasive cervical cancer: a retrospective cross-sectional worldwide study. Lancet Oncol..

[22] Kiernan J. Staining theory. Histol Histochem Methods. 2008;

[23] Félix A, Alemany L, Tous S, de Sanjosé S, Bosch FX (2016). HPV distribution in cervical cancer in Portugal. A retrospective study from 1928 to 2005. Papillomavirus Res..

[24] Larsson GL, Carlsson J, Karlsson MG, Helenius G (2015). Evaluation of HPV genotyping assays for archival clinical samples. J Mol Diagnost.

[25] Alvarez-Aldana A, Martínez JW, Sepúlveda-Arias JC (2015). Comparison of five protocols to extract DNA from paraffin-embedded tissues for the detection of human papillomavirus. Pathol Res Pract..

[26] Mengual-Chuliá B, Bedhomme S, Lafforgue G, Elena SF, Bravo IG (2016). Assessing parallel gene histories in viral genomes. BMC Evol Biol..

[27] Baay MF, Quint WG, Koudstaal J, Hollema H, Duk JM, Burger MP (1996). Comprehensive study of several general and type-specific primer pairs for detection of human papillomavirus DNA by PCR in paraffin-embedded cervical carcinomas. J Clin Microbiol..

[28] de Roda Husman A-M, Walboomers JMM, van den Brule AJC, Meijer CJLM, Snijders PJF (1995). The use of general primers GP5 and GP6 elongated at their 3’ ends with adjacent highly conserved sequences improves human papillomavirus detection by PCR. J Gen Virol..

[29] Nakayama Y, Yamaguchi H, Einaga N, Esumi M (2016). Pitfalls of DNA quantification using dnabinding fluorescent dyes and suggested solutions. PLoS One..

[30] Herraez-Hernandez E, Alvarez-Perez M, Navarro-Bustos G, Esquivias J, Alonso S, Aneiros-Fernandez J (2013). HPV Direct Flow CHIP: a new human papillomavirus genotyping method based on direct PCR from crude-cell extracts. J Virol Methods..

[31] Martró E, Valencia MJ, Tarrats A, Castellà E, Llatjós M, Franquesa S (2012). Comparison between two human papillomavirus genotyping assays targeting the L1 or E6/E7 region in cervical cancer biopsies. Enferm Infecc Microbiol Clin [Internet]..

[32] Dal Bello B, Spinillo A, Alberizzi P, Cesari S, Gardella B, Silini EM (2009). Time trends of human papillomavirus type distribution in Italian women with cervical intraepithelial neoplasia (CIN). Gynecol Oncol [Internet]..

