# Supplementary material for: Human DNA decays faster with time than viral dsDNA: an analysis on HPV16 using pathology archive samples spanning 85 years
Source: Virol J. 2021 Mar 29;18:65. doi: 10.1186/s12985-021-01529-9 (PMC8008572; doi:10.1186/s12985-021-01529-9)
Supplement: Supplementary file 1 — Additional file1 Fig S1. Violin plots comparing the distribution of the quantification values by gene, amplicon size and storage time including all samples. For each period of time, median values of copies/µL (depicted as Log10) per amplicon either between host and virus or for the two different amplicons within host and within viruses are compared by means of a Wilcoxon Mann–Whitney test. For each comparison, p-values for the null-hypothesis of non-different median values between the corresponding distributions are indicated. Fragments are represented as follows: “Human short” to represent 65 bp tubulin-β gene amplicon; “Human long” to represent 149 bp tubulin-β gene amplicon; “Viral short” to represent 69 bp L1 gene amplicon and “Viral long” to represent 134 bp L1 gene amplicon. Each period of time is represented below fragments. [file 12985_2021_1529_MOESM1_ESM.docx]

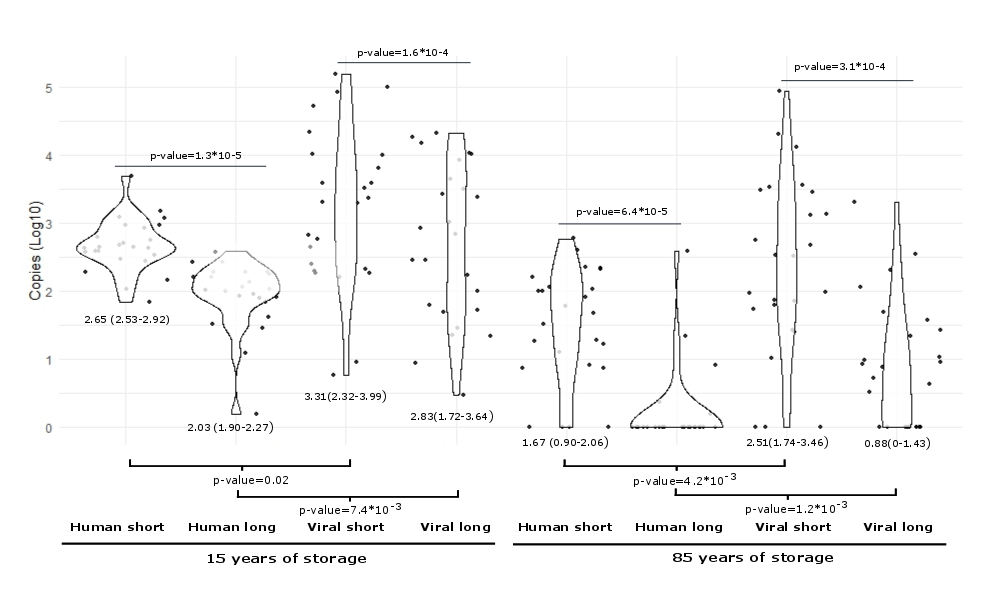
**Figure S1.** Violin plots comparing the distribution of the values of quantification by gene and amplicon size in the two periods of storage.

Median number of copies/µL in Log_10_ are compared *per* fragment for each period of time through Wilcoxon Mann-Whitney test. H_0_ should be read as follows Copies in Log_10_ being similar in both fragment comparison for each period of time is rejected with p-value below 0.05.

Fragments are represented as follow: “Human short” to represent 65 bp *Tubulin*-β gene amplicon; “Human long” to represent 149 bp *Tubulin*-β gene amplicon; “Viral short” to represent 69 bp *L1* gene amplicon and “Viral long” to represent 134 bp *L1* gene amplicon. Each period of time is represented below fragments.
